# Whole Genome Sequencing, *de Novo* Assembly and Phenotypic Profiling for the New Budding Yeast Species *Saccharomyces jurei*

**DOI:** 10.1534/g3.118.200476

**Published:** 2018-08-14

**Authors:** Samina Naseeb, Haya Alsammar, Tim Burgis, Ian Donaldson, Norman Knyazev, Christopher Knight, Daniela Delneri

**Affiliations:** Manchester Institute of Biotechnology, The University of Manchester, UK, M1 7DN

**Keywords:** evolution, fitness, PacBio, translocation, *Saccharomyces*

## Abstract

*Saccharomyces sensu stricto* complex consist of yeast species, which are not only important in the fermentation industry but are also model systems for genomic and ecological analysis. Here, we present the complete genome assemblies of *Saccharomyces jurei*, a newly discovered *Saccharomyces sensu stricto* species from high altitude oaks. Phylogenetic and phenotypic analysis revealed that *S. jurei* is more closely related to *S. mikatae*, than *S. cerevisiae*, and *S. paradoxus*. The karyotype of *S. jurei* presents two reciprocal chromosomal translocations between chromosome VI/VII and I/XIII when compared to the *S. cerevisiae* genome. Interestingly, while the rearrangement I/XIII is unique to *S. jurei*, the other is in common with *S. mikatae* strain IFO1815, suggesting shared evolutionary history of this species after the split between *S. cerevisiae* and *S. mikatae*. The number of Ty elements differed in the new species, with a higher number of Ty elements present in *S. jurei* than in *S. cerevisiae*. Phenotypically, the *S. jurei* strain NCYC 3962 has relatively higher fitness than the other strain NCYC 3947^T^ under most of the environmental stress conditions tested and showed remarkably increased fitness in higher concentration of acetic acid compared to the other *sensu stricto* species. Both strains were found to be better adapted to lower temperatures compared to *S. cerevisiae*.

*Saccharomyces sensu stricto* yeasts, currently comprise eight species: *S*. *cerevisiae*, *S*. *paradoxus*, *S*. *uvarum*, *S*. *mikatae*, *S*. *kudriavzevii*, *S. arboricola*, *S*. *eubayanus*, *S. jurei* (Martini and Martini 1987; Wang and Bai 2008; Naumov *et al.* 2000; Naumov *et al.* 1995a; Naumov *et al.* 1995b; Libkind *et al.* 2011; Naseeb *et al.* 2017b) and two natural hybrids: *S. pastorianus* (Masneuf *et al.* 1998; Querol and Bond 2009) and *S. bayanus* (Nguyen *et al.* 2011). *Saccharomyces jurei* is the latest addition to the *sensu stricto* clade and was isolated from oak tree bark and surrounding soil at an altitude of 1000 m above sea level in Saint Auban, France (Naseeb *et al.* 2017b). It is known that species within the *sensu stricto* group are reproductively isolated and possess post- zygotic barriers (Naumov 1987). Moreover, yeasts within this group exhibit almost identical karyotypes with 16 chromosomes (Cardinali and Martini 1994; Carle and Olson 1985; Naumov *et al.* 1996).

In the modern era of yeast genetics, the advances in sequencing technology have lead to the whole genome sequencing of many *Saccharomyces sensu stricto* species (*S. cerevisiae*, *S. bayanus var. uvarum*, *S. kudriavzevii*, *S. mikatae*, *S. paradoxus*, *S. eubayanus* and *S. arboricola*) (Libkind *et al.* 2011; Liti *et al.* 2013; Cliften *et al.* 2003; Kellis *et al.* 2003; Scannell *et al.* 2011; Casaregola *et al.* 2000). To date, more than 1000 *S. cerevisiae* strains belonging to different geographical and environmental origins have been sequenced and assembled (Engel and Cherry 2013; Peter *et al.* 2018). The availability of sequencing data from multiple strains of Saccharomycotina yeast species has enhanced our understanding of biological mechanisms and comparative genomics. Researchers are now combining comparative genomics with population ecology to better understand the genetic variations, taxonomy, evolution and speciation of yeast strains in nature. Genome variation provides the raw material for evolution, and may arise by various mechanisms including gene duplication, horizontal gene transfer, hybridization and micro and macro rearrangements (Fischer *et al.* 2001; Seoighe *et al.* 2000; Lynch 2002; Hall *et al.* 2005; Naseeb *et al.* 2017a; Naseeb *et al.* 2016; Naseeb and Delneri 2012). Synteny conservation studies have shown highly variable rates of genetic rearrangements between individual lineages both in vertebrates and in yeasts (Bourque *et al.* 2005; Fischer *et al.* 2006; Vakirlis *et al.* 2016). This genome variation is a means of evolutionary adaptation to environmental changes. An understanding of the genetic machinery linked to phenotypic variation provides knowledge of the distribution of *Saccharomyces* species in different environments, and their ability to withstand specific conditions (Goddard and Greig 2015; Jouhten *et al.* 2016; Brice *et al.* 2018; Peter *et al.* 2018).

Recently, we isolated two strains (NCYC 3947^T^ and NCYC 3962) of *Saccharomyces jurei* from *Quercus robur* bark and surrounding soil (Naseeb *et al.* 2017b). The initial sequencing of ITS1, D1/D2 and seven other nuclear genes showed that both strains of *S. jurei* were closely related to *S. mikatae* and *S. paradoxus* and grouped in *Saccharomyces sensu stricto* complex. We also showed that *S. jurei* can readily hybridize with other *sensu stricto* species but the resulting hybrids were sterile (Naseeb *et al.* 2017b). Here, we represent high quality *de novo* sequence and assembly of both strains (NCYC 3947^T^ and NCYC 3962) of *S. jurei*. The phylogenetic analysis placed *S. jurei* in the *sensu stricto* clade, in a small monophyletic group with *S. mikatae*. By combining Illumina HiSeq and PacBio data, we were able to assemble full chromosomes and carry out synteny analysis. Moreover, we show that *S. jurei* NCYC 3962 had higher fitness compared to NCYC 3947^T^ under different environmental conditions. Fitness of *S. jurei* strains at different temperatures showed that it was able to grow at wider range of temperatures (12°-37°).

## Material and Methods

### Yeast strains

Strains used in this study are presented in Table 1. All strains were grown and maintained on YPDA (1% w/v yeast extract, 2% w/v Bacto-peptone, 2% v/v glucose and 2% w/v agar). Species names and strains number are stated in Table 1.

**Table 1:** Strains used in this study

Species	Strain number	References
*S. jurei*	NCYC 3947^T^	(Naseeb *et al.* 2017b)
	NCYC 3962	
*S. cerevisiae*	NCYC 505^T^	(Vaughan Martini and Kurtzman 1985)
*S. paradoxus*	CBS 432^T^	(Naumov 1987)
*S. mikatae*	NCYC 2888^T^ (IFO 1815^T^)	(Yamada *et al.* 1993)
*S. kudriavzevii*	NCYC 2889^T^ (IFO 1802^T^)	(Yamada *et al.* 1993)
*S. arboricola*	CBS 10644^T^	(Wang and Bai 2008)
*S. eubayanus*	PYCC 6148^T^ (CBS 12357^T^)	(Libkind *et al.* 2011)
*S. uvarum*	NCYC 2669 (CBS 7001)	(Pulvirenti *et al.* 2000)
*S. pastorianus*	NCYC 329^T^ (CBS 1538^T^)	(Martini and Martini 1987)

### DNA Extraction

For Illumina Hiseq, the total DNA was extracted from an overnight grown culture of yeast strains by using the standard phenol/chloroform method described previously (Fujita and Hashimoto 2000) with some modifications. Briefly, 5 ml of overnight grown yeast cells were centrifuged and resuspended in 500 μl EB buffer (4M sorbitol, 500mM EDTA and1M DTT) containing 1 mg/ml lyticase. The cells were incubated at 37° for 1 hr. Following incubation, the cells were mixed with stop solution (3M NaCl, 100mM Tris pH 7.5 and 20mM EDTA) and 60 μl of 10% SDS. The cell suspension was vortexed and mixed with 500 μl phenol-chloroform. The samples were centrifuged at 13000 rpm for 2 min to separate the aqueous phase from the organic phase. The upper aqueous phase was transferred to a clean 1.5 ml tube and phenol-chloroform step was repeated twice until a white interface was no longer present. The aqueous phase was washed with 1 ml absolute ethanol by centrifugation at 13000 rpm for 10 min. The pellet was air dried and resuspended in 30 μl of sterile milliQ water.

Genomic DNA for PacBio sequencing was extracted using Qiagen Genomic-tip 20/G kit (cat. No. 10223) following manufacturer’s recommended instructions. The yield of all DNA samples was assessed by the nanodrop spectrophotometer (ND-1000) and by Qubit 2.0 fluorometer (catalog no. Q32866). Purity and integrity of DNA was checked by electrophoresis on 0.8% (w/v) agarose gel and by calculating the A260/A280 ratios.

### Library preparation for Illumina and PacBio sequencing

Paired end whole-genome sequencing was performed using the Illumina HiSeq platform. FastQC (Babraham Bioinformatics) was used to apply quality control to sequence reads, alignment of the reads was performed using BOWTIE2 (Langmead and Salzberg 2012) and post-processed using SAMTOOLS (Li *et al.* 2009).

For Pacbio sequencing, genomic DNA (10 μg) of NCYC 3947^T^ and NCYC 3962 strains was first DNA damage repaired, sheared with Covaris G-tube, end repaired and exonuclease treated. SMRTbell library (10-20kb size) was prepared by ligation of hairpin adaptors at both ends according to PacBio recommended procedure (Pacific Bioscience, No: 100-259-100). The resulting library was then size selected using Blue Pippin with 7-10kb cut-off. Sequencing run was performed on PacBio RS II using P6/C4 chemistry for 4 hr. The genome was assembled using SMRT analysis and HGAP3 pipeline was made using default settings.

### Genome assembly, annotation, orthology and chromosomal structural plots

The PacBio sequences were assembled using hierarchical genome-assembly process (HGAP) (Chin *et al.* 2013). Protein coding gene models were predicted using Augustus (Stanke and Morgenstern 2005) and the Yeast Genome Annotation Pipeline (Byrne and Wolfe 2005). In addition, protein sequences from other *Saccharomyces* species were aligned to the genome assembly using tblastn (Gertz *et al.* 2006). These predictions and alignments were used to produce a final set of annotated genes with the Apollo annotation tool (Lewis *et al.* 2002). The protein sequences were functionally annotated using InterproScan (Jones *et al.* 2014). Orthologous relationships with *S. cerevisiae* S288C sequences were calculated using InParanoid (Berglund *et al.* 2008). Non-coding RNAs were annotated by searching the RFAM database (Nawrocki *et al.* 2015) using Infernal (Nawrocki and Eddy 2013). Further tRNA predictions were produced using tRNAscan (Lowe and Eddy 1997). Repeat sequences were identified in Repbase (Bao *et al.* 2015) using Repeat Masker (Smit *et al.* 2013–2015). The dotplots were constructed by aligning *S. jurei* genome to the *S. cerevisiae* S288C genome using NUCmer and plotted using MUMmerplot (Kurtz *et al.* 2004). These features are available to browse via a UCSC genome browser (Kent *et al.* 2002) track hub (Raney *et al.* 2014). Single nucleotide polymorphisms (SNPs) were identified using Atlas-SNP2(Challis *et al.* 2012).

### Phenotypic assays

#### Temperature tolerance:

Fitness of *S. jurei* strains and *Saccharomyces sensu stricto* type strains was examined using FLUOstar optima microplate reader at 12°, 16°, 20°, 25°, 30° and 37°. Cells were grown from a starting optical density (OD) of 0.15 to stationary phase in YPD (1% w/v yeast extract, 2% w/v Bacto-peptone and 2% w/v glucose) medium. The growth OD_595_ was measured every 5 min with 1 min shaking for 72 hr. Growth parameters, lag phase (***λ***), maximum growth rate (µ_max_), and maximum biomass (*A*max) were estimated using R shiny app on growth curve analysis (https://kobchai-shinyapps01.shinyapps.io/growth_curve_analysis/).

#### Environmental stress:

Strains were screened for tolerance to environmental stressors using a high-throughput spot assay method. Cells were grown in a 96-well plate containing 100 µl YPD in four replicates at 30° for 48 hr. The yeast strains grown in 96-well plate were sub-cultured to a 384 well plate to achieve 16 replicates of each strain and grown at 30° for 48 hr. Singer ROTOR HDA robot (Singer Instruments, UK) was used to spot the strains on (i) YPDA + 0.4% & 0.6% acetic acid, (ii) YPDA+ 4mM & 6mM H_2_O_2_, (iii) YPDA+ 2.5mM & 5mM CuSO_4_, (iv) YPDA+ 2% & 5% NaCl, (v) YPDA+ 5% & 10% Ethanol (vi) YPA+ 15% maltose and (vii) YPA+ 30% & 35% glucose_._ The spot assay plates were incubated at 30° and high-resolution images of phenotypic plates were taken using phenobooth after 3 days of incubation (Singer Instruments, UK). The colony sizes were calculated in pixels using phenosuite software (Singer Instruments, UK) and the heat maps of the phenotypic behaviors were constructed using R shiny app (https://kobchai-shinyapps01.shinyapps.io/heatmap_construction/).

### Data and reagent availability

Strains are available upon request. Supplemental files are available at FigShare (https://figshare.com/s/60bbbc1e98886077182a). Figure S1 shows alignment of the amino acid sequences of *MEL1* gene belonging to *S. jurei* NCYC 3947^T^ (Sj) and *S. mikatae* IFO 1816 (Sm). Table S1, Table S2, Table S3 and Table S4 list the genes, which are present in simple one to one orthologous relationship, in many to many relationship, in many to one relationship and in one to many relationship, respectively. Table S5 lists the genes that are present in *S. cerevisiae* but absent in *S. jurei*. Table S6 lists the genes which are present in *S. jurei* but absent in *S. cerevisiae*. Table S7 lists the genes which are used to construct the phylogenetic tree. Table S8 lists the genes which are potentially introgressed in *S. jurei* genome from *S. paradoxus*. Table S9, Table S10 and Table S11 show lag phase time (*λ*), maximum growth rate (µmax) and maximum biomass (*A*_max_) of *Saccharomyces species* used in this study, respectively. The sequences and annotations reported in this paper are available in the European Nucleotide Archive under project ID PRJEB24816, assembly ID GCA_900290405 and accession number ERZ491603.

## Results and Discussion

### High quality de novo sequencing and assembly of S. jurei genome

Genome sequencing of the diploid *S. jurei* NCYC 3947^T^ and NCYC 3962 yeast strains was performed using Illumina Hiseq and Pacbio platforms. We obtained approximately 9.02 × 10^5^ and 4.5 × 10^5^ reads for NCYC 3947^T^ and NCYC 3962 respectively. We obtained 2 × 101 bp reads derived from ∼200 bp paired-end reads which were assembled in 12 Mb genome resulting in a total coverage of 250x based on high quality reads. The sequencing results and assembled contigs are summarized in Tables 2, 3, and 4. By combining the Illumina mate pair and Pacbio sequencing we were able to assemble full chromosomes of *S. jurei* NCYC 3947^T^ and NCYC 3962 (Tables 5 and 6). The total genome size (∼12 Mb) obtained for both strains of *S. jurei* was comparable to the previously published genomes of *Saccharomyces sensu stricto* species (Scannell *et al.* 2011; Goffeau *et al.* 1996; Liti *et al.* 2013; Baker *et al.* 2015).

**Table 2:** Summary of *S. jurei* NCYC 3947^T^ genome sequencing and assembly using Hi-seq platform

Metric	Contigs	Contigs >= 500bp	Scaffolds	Scaffolds >= 500bp
Number	810	250	753	211
Total Length	11,938,007	11,869,594	11,940,421	11,869,594
Length Range	87-673,524	525-673,524	87-673,524	525-673,524
Average Length	14,738	56,254	15,857	56,254
N50	172,207	279,631	279,631	279,631

**Table 3:** Summary of *S. jurei* NCYC 3962 genome sequencing and assembly using Hi-seq platform

Metric	Contigs	Contigs >= 500bp	Scaffolds	Scaffold >= 500bp
Number	3719	987	3618	933
Total length	11,760,925	11,419,281	11,768,034	11,441,494
Length range	59-80,684	507-80,684	59-80,684	507-80,684
Average length	3,162	11,569	3,252	12,263
N50	20,806	21,318	21,928	22,552

**Table 4:** Summary of *S. jurei* NCYC 3947^T^ and NCYC 3962 genome assembly using PacBio platform

Metric	*S. jurei* NCYC 3947	*S. jurei* NCYC 3962
Contigs	35	57
Max contig length	1,474,466	1,470,125
Contig N50	738,741	652,030
Total assembly size	12,306,756	12,932,708

**Table 5:** Total lengths of chromosomes assembled in *S. jurei* NCYC 3947^T^

Sequence name	Length (bp) including gaps
chrI.1_chrXIII.2	809,572
chrII	809,280
chrIII	308,350
chrIV	1,474,466
chrV	584,553
chrVI.1_chrVII.2	730,011
chrVI.2_chrVII.1	638,210
chrVIII	534,462
chrIX	434,517
chrX	738,741
chrXI	671,067
chrXII.1	458,950
chrXII.2	568,540
chrI.2_chrXIII.1	334,136
chrXIV	749,072
chrXV	1,068,672
chrXVI	920,427
chrMT	105,732

**Table 6:** Total lengths of chromosomes assembled in *S. jurei* NCYC 3962

Sequence name	Length (bp) including gaps
chrI.1_chrXIII.2	756,315
chrII	814,183
chrIII	329,028
chrIV	1,470,125
chrV	570,437
chrVI.1_chrVII.2	723,619
chrVII.2_chrVI.1	652,030
chrVIII	536,516
chrIX	439,662
chrX.1	487,336
chrX.2	258,684
chrXI	676,065
chrXII.1	475,978
chrXII.2	571,082
chrI.2_chrXIII.1	334,998
chrXIV	790,124
chrXV.1	474,048
chrXV.2	240,703
chrXV.3	236,823
chrXV.4	114,889
chrXVI	806,586
chrMT	110,829

### S. jurei genome prediction and annotation

The high-quality *de novo* assembly of *S. jurei* NCYC 3947^T^ genome resulted in 5,794 predicted protein-coding genes for *S. jurei*, which is similar to the published genomes of other *sensu stricto* species (Baker *et al.* 2015; Liti *et al.* 2009; Liti *et al.* 2013; Scannell *et al.* 2011; Walther *et al.* 2014). Of the predicted protein-coding genes, 5,124 were in a simple 1:1 putatively orthologous relationship between *S. cerevisiae* and *S. jurei* (Table S1). From the remaining protein-coding genes, 35 genes showed many to many relationship (multiple *S. cerevisiae* genes in paralogous cluster with multiple *S. jurei* genes (Table S2), 31 genes were in many to one relationship (many genes in *S. cerevisiae* are in an paralogous cluster with a single *S. jurei* gene; most of these were found to be retrotransposons; Table S3) and 50 genes were in one to many relationships (one *S. cerevisiae* gene in an paralogous cluster with many *S. jurei* genes; Table S4). Interestingly, we found an increase in the copy number of maltose metabolism and transport genes (*IMA1*, *IMA5*, *MAL31*, and *YPR196W*- 2 copies of each gene), flocculation related gene (*FLO1*- 2 copies) and hexose transporter (*HXT8*- 3 copies). Increased dosage of these genes in *S. jurei* could have conferred selective advantage toward better sugar utilization (Lin and Li 2011; Ozcan and Johnston 1999; Soares 2011; Adamczyk *et al.* 2016). Genes encoding for PAU proteins (a member of the seripauperin multigene family), copper resistance and salt tolerance related genes were found to be present in fewer copies in *S. jurei* genome compared to *S. cerevisiae*. This variation in copy number of genes in a genome can have phenotypic and physiological effects on the species (Landry *et al.* 2006; Adamo *et al.* 2012; Gorter de Vries *et al.* 2017).

We also searched for the presence of repetitive elements in *S. jurei* NCYC 3947^T^ and NCYC 3962 using BLAST and compared them to the Ty elements in *S. cerevisiae*. We detected Ty1-LTR, Ty2-LTR, Ty2-I-int, Ty3-LTR, Ty3-I and Ty4 sequences in both strains of *S. jurei*. Interestingly, we found an increased number of Ty1-LTR, Ty2-LTR, Ty3-LTR and Ty4 elements in *S. jurei* genome compared to *S. cerevisiae* (Table 7). High copy numbers of Ty1, Ty2, and Ty3 transposable elements have also been reported in different strains of *S. cerevisiae*, *e.g.*, Ty1 and Ty2 in French Dairy, Ty2 in Alpechin, Ty1 in Mexican Agave, and Ty3 in Ecuadorean clade (Peter *et al.* 2018; Bleykasten-Grosshans *et al.* 2013). Repetitive sequences are found in genomes of all eukaryotes and can be a potential source of genomic instability since they can recombine and cause chromosomal rearrangements, such as translocations, inversions and deletions (Naseeb *et al.* 2016; Shibata *et al.* 2009; Chan and Kolodner 2011).

**Table 7:** Counts of Ty elements in *S. cerevisiae*, *S. jurei* NCYC 3947^T^ and NCYC 3962

Ty elements	Ty elements annotation	Counts in *S. cerevisiae*	Counts in *S. jurei* NCYC 3947^T^	Counts in *S. jurei* NCYC 3962
Ty	Yeast Ty transposable element Ty-pY109 near tRNA-Lys1 gene	164	71	74
Ty1-LTR	Ty1 LTR-retrotransposon from yeast (LTR)	124	276	272
Ty2-LTR	Ty2 LTR-retrotransposon from yeast (LTR)	108	118	117
Ty2-I-int	Ty2 LTR-retrotransposon from yeast (internal portion).	15	2	2
Ty3-LTR	*S. paradoxus* Ty3-like retrotransposon, Long terminal repeat	61	70	71
Ty3-I	*S. paradoxus* Ty3-like retrotransposon, Internal region.	2	1	1
Ty4	Gag homolog, Ty4B = protease, integrase, reverse transcriptase,and RNase H domain containing protein {retrotransposon Ty4}	51	164	162

### *Saccharomyces jurei* share a chromosomal translocation With *Saccharomyces mikatae* IFO 1815

**Figure 1:**
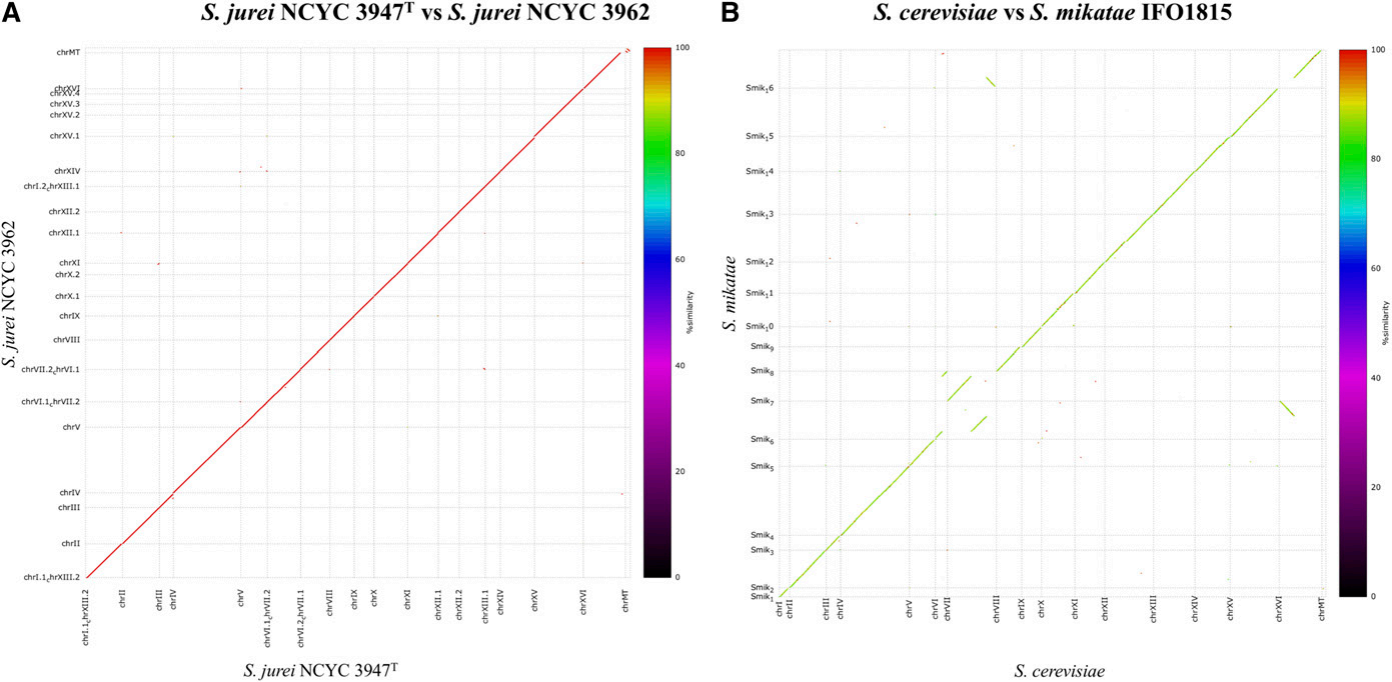
Dot plot alignments comparing the chromosome sequence identity of *S. jurei* NCYC 3947^T^ *vs. S. jurei* NCYC 3962 (A) and *S. cerevisiae* S288C *vs. S. mikatae* IFO1815 (B). The broken lines represent chromosomal translocations between chromosomes VI / VII and XVI / VII.

**Figure 2:**
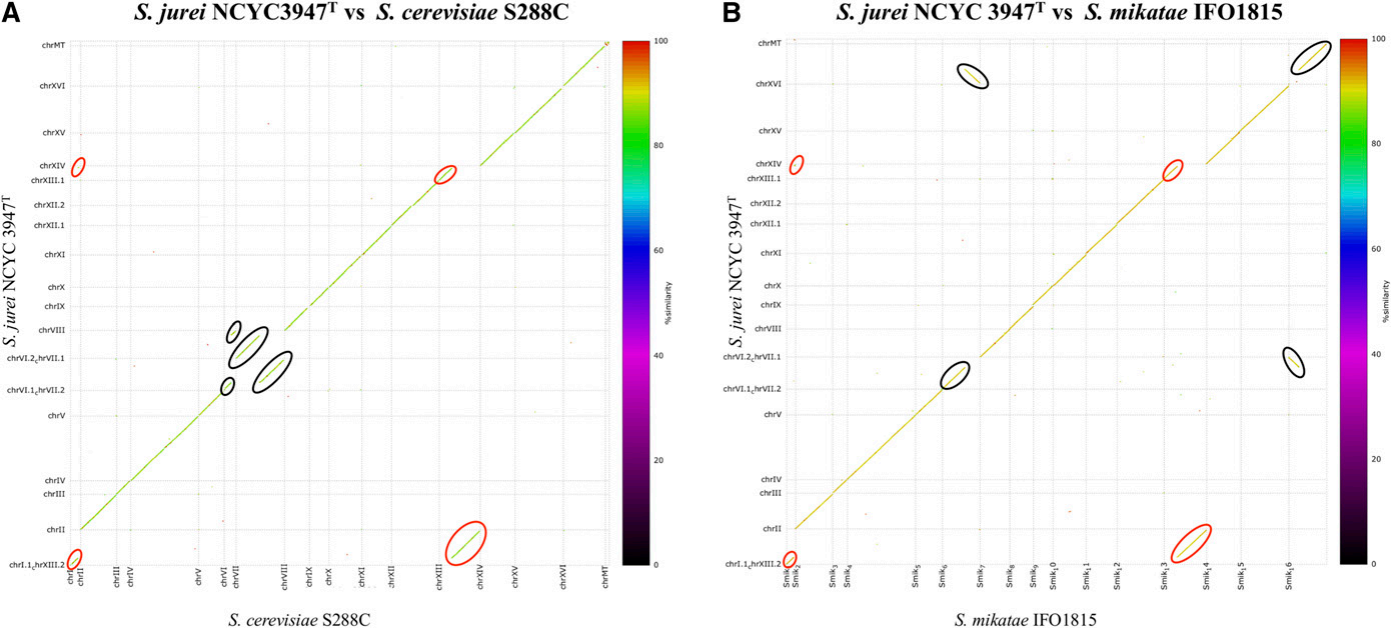
Dot plot alignments comparing the chromosome sequence identity of *S. jurei* NCYC 3947^T^ *vs. S. cerevisiae* S288C (A) and *S. jurei* NCYC 3947^T^ *vs. S. mikatae* IFO1815 (B). Black ovals represent the translocation between chromosomes VI and VII, which is common in *S. mikatae* and *S. jurei* whereas red ovals represent the translocation between chromosomes I and XIII, which is unique to *S. jurei*.

To check the presence or absence of genomic rearrangements in *S. jurei*, we compared the chromosome structures between *S. jurei* NCYC 3947^T^ and *S. jurei* NCYC 3962 (Figure 1A), between *S. cerevisiae* S288C and *S. mikatae* IFO1815 (Figure 1B), between *S. jurei* NCYC 3947^T^ and *S. cerevisiae* S288C (Figure 2A) and between *S. jurei* NCYC 3947^T^ and *S. mikatae* IFO1815 (Figure 2B). The two *S. jurei* strains had a syntenic genome (Figure 1A), while we identified two chromosomal translocations with *S. cerevisiae* S288C (Figure 2A). One translocation is unique to *S. jurei* and is located between chromosomes I and XIII (Figure 2, red ovals), while the second translocation is located between chromosomes VI and VII in the same position of the previously identified translocation in *S. mikatae* IFO1815 (Figure 2, black ovals).

The breakpoints of the translocation I/XIII are in the intergenic regions between uncharacterized genes. The breakpoints neighborhood is surrounded by several Ty elements (Ty1-LTR, Ty4, and Ty2-LTR) and one tRNA, which may have caused the rearrangement (Bridier-Nahmias *et al.* 2015; Fischer *et al.* 2000; Liti *et al.* 2013; Mieczkowski *et al.* 2006). The translocation in common with *S. mikatae* shares the same breakpoints between open reading frames (ORFs) YFR006W and YFR009W on chromosome VI, and between ORFs YGR021W and YGR026W on chromosome VII. This translocation is also shared by both strains of *S. mikatae*, but not with other *Saccharomyces sensu stricto* species. Overall this suggests a common evolutionary history between these strains and species, however an adaptive value of this rearrangement or a case of breakpoint re-usage cannot be ruled out since rearrangements can be adaptive with evidence both from nature and lab setting. (Chang *et al.* 2013; Dunham *et al.* 2002; Avelar *et al.* 2013; Colson *et al.* 2004; Adams *et al.* 1992; Fraser *et al.* 2005; Hewitt *et al.* 2014). Several natural isolates of *S. cerevisiae* present karyotypic changes (Hou *et al.* 2014) and the reciprocal translocation present between chromosomes VIII and XVI is able to confer sulphite resistance to the yeasts strains in vineyards (Perez-Ortin *et al.* 2002). Furthermore, lab experimental evolution studies in different strains of *S. cerevisiae* when evolved under similar condition end up sharing the same breakpoints (Dunham *et al.* 2002). Previous studies on mammalian systems have shown that breakpoints maybe reused throughout evolution at variable rates (Larkin *et al.* 2009; Murphy *et al.* 2005), and breakpoint re-usage has also been found between different strains of *S. pastorianus* (Hewitt *et al.* 2014).

### Novel genes present in S. jurei

The comparison between *S. jurei* and *S. cerevisiae* genome showed 622 differentially present genes. 179 open reading frames (ORFs) were predicted to be novel in *S. jurei* when compared to *S. cerevisiae* reference S288C strain (Table S5). To further confirm if these ORFs were truly novel, we analyzed the sequences in NCBI nucleotide database and in *Saccharomyces* Genome Database (SGD) against all the fungal species. We found 4 novel ORFs that have no significant match to any of the available genomes (Table S5-shown in red). 5 ORFs gave partial similarity to different fungal species such as *Vanderwaltozyma polyspora*, *Kluyveromyces marxianus*, *Torulaspora delbrueckii*, *Zygosaccharomyces rouxii*, *Hyphopichia burtonii*, *Kazachstania africana*, *Trichocera brevicornis*, *Lachancea walti*, and *Naumovozyma castellii* (Table S5-yellow highlighted). Majority of the remaining sequences gave full or partial matches to *S. cerevisiae* natural isolates (Strope *et al.* 2015; Peter *et al.* 2018), *S. paradoxus*, *S. mikatae*, *S. kudriavzevii*, *S. bayanus*, *S. uvarum*, and *S. eubayanus*.

Moreover, we also found 462 ORFs, which are present in *S. cerevisiae* genome but were lost in *S. jurei* (Table S6). The Gene Ontology (GO) analysis of these genes showed significant enrichment of RNA-directed DNA polymerase activity, aryl-alcohol dehydrogenase (NAD+) activity, DNA-directed DNA polymerase activity, and asparaginase activity. The majority of genes which were novel or lost in *S. jurei* were found to be subtelomeric or telomeric, in regions known to show higher genetic variations (Bergström *et al.* 2014).

The genes lost in *S. jurei* encompass functionally verified ORFs, putative genes and uncharacterized genes. Some of the verified ORFs included ribosomal subunits genes, asparagine catabolism genes, alcohol dehydrogenase genes, hexose transporters, genes involved in providing resistance to arsenic compounds, phosphopyruvate hydratase genes, iron transport facilitators, ferric reductase genes and flocculation related genes.

**Figure 3:**
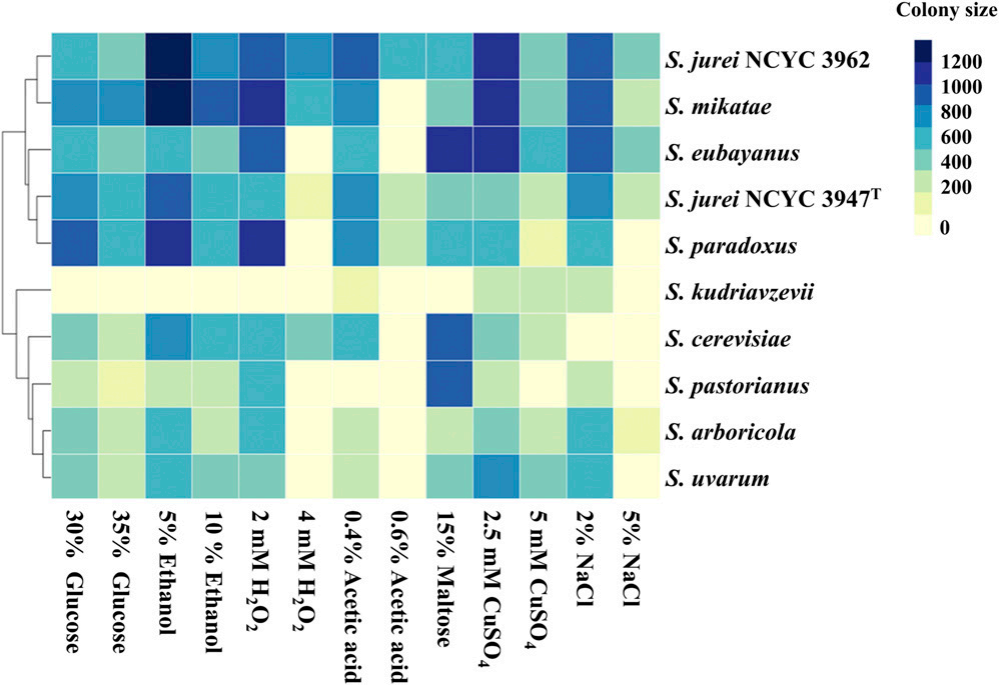
Heat map representing phenotypic fitness of *S. jurei* NCYC 3947^T^ and NCYC 3962 compared to *sensu stricto* species type strains in response to different environmental stressors at 30°C. Phenotypes are represented with colony sizes calculated as pixels and colored according to the scale, with light yellow and dark blue colors representing the lowest and highest growth respectively. Hierarchical clustering of the strains is based on the overall growth profile under different media conditions tested.

We found that *S. jurei* genome lacks four out of seven alcohol dehydrogenase (AAD) genes including the functional *AAD4* gene, which is involved in oxidative stress response (Delneri *et al.* 1999a; Delneri *et al.* 1999b). Although *S. jurei* has lost *AAD4* gene, however, it was able to tolerate oxidative stress caused by 4mM H_2_O_2_ (Figure 3A).

All four genes of the *ASP3* gene cluster located on chromosome XII are absent in *S. jurei*. It was not surprising since this gene cluster is only known to be present in *S. cerevisiae* strains isolated from industrial and laboratory environments and lost from 128 diverse fungal species (Gordon *et al.* 2009; League *et al.* 2012). These genes are up-regulated during nitrogen starvation allowing the cells to grow by utilizing extracellular asparagine as a nitrogen source.

The hexose transporter family consists of 20 putative HXT genes (*HXT1-HXT17*, *GAL2*, *SNF3*, and *RGT2*) located on different chromosomes (Boles and Hollenberg 1997; Kruckeberg 1996) of which *HXT15*, *HXT16* and *HXT2* are absent from *S. jurei*. Under normal conditions, only 6 HXT genes (*HXT1* and *HXT3-HXT7*) are known to play role in glucose uptake suggesting that loss of 3 HXT genes from *S. jurei* is unlikely to affect glucose transport (Lin and Li 2011).

### Heterozygosis and strain divergence in the *S. jurei*

To detect genetic divergence between the two strains we mapped SNPs between the strains (NCYC 3947^T^ *vs.* NCYC 3962), while to detect heterozygosis, we mapped the Single Nucleotide Polymorphisms (SNPs) in the two sets of alleles within the novel strains (NCYC 3947^T^ *vs.* NCYC 3947^T^, and NCYC 3962 *vs.* NCYC 3962). We found 6227 SNPs between the two strains, showing a genetic divergence between them, which is relatively lower compared to the genetic divergence found among *S. cerevisiae* strains. Moreover, 278 and 245 SNPs were found within NCYC 3947^T^ and NCYC 3962 strains respectively, indicating a low level of heterozygosity within each strain (Table 8). 139 SNPs were found be to common to both strains. Previous studies on *S. cerevisiae* and *S. paradoxus* strains from different lineages have shown that the level of heterozygosity is variable, with a large number of strains showing high level of heterozygosity isolated from human associated environments (Magwene *et al.* 2011; Tsai *et al.* 2008). A more recent study on 1011 *S. cerevisiae* natural strains showed that 63% of the sequenced isolates were heterozygous (Peter *et al.* 2018).

**Table 8:** Approximate numbers of SNPs in *S. jurei* NCYC 3947^T^ and NCYC 3962 genome

Reference genome	Genome mapped	SNPs
NCYC 3947^T^	NCYC 3947^T^	278
NCYC 3962	NCYC 3962	245
NCYC 3947^T^	NCYC 3962	5702
NCYC 3962	NCYC 3947^T^	6227

### Phylogenetic analysis

**Figure 4:**
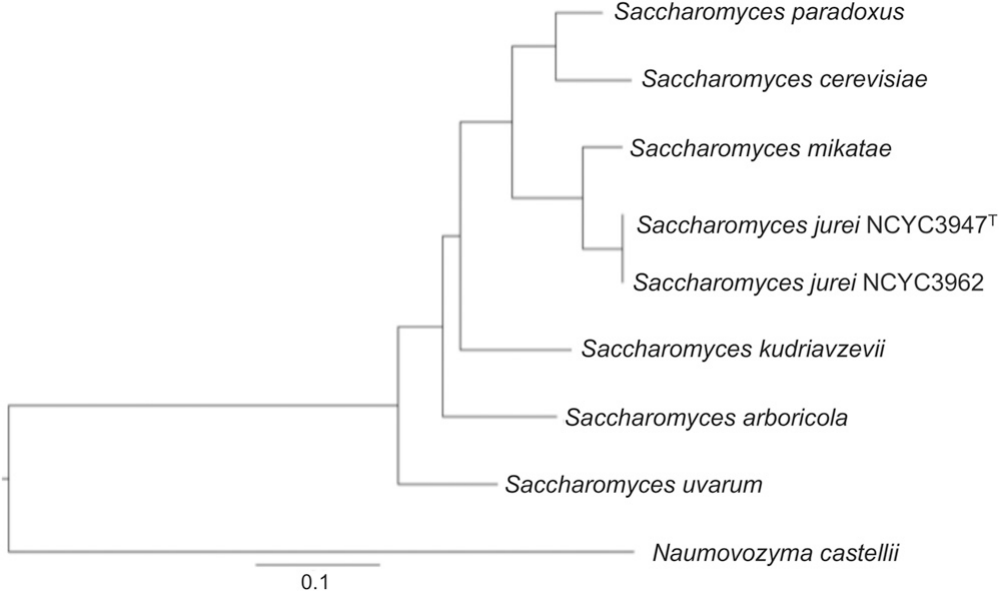
Phylogenetic tree showing both novel strains located in one single monophyletic group, with the *S. mikatae*. Maximum likelihood phylogeny was constructed using a concatenated alignment of 101 universally distributed genes. Sequences from all *Saccharomyces sensu stricto* species were aligned using StatAlign v3.1 and phylogenetic tree was built using RaxML 8.1.3 with *N. castellii* kept as out-group.

A first phylogeny construction using ITS/D1+D2 sequence analysis showed that *S. jurei* is placed in the tree close to *S. cerevisiae*, *S. mikatae* and *S. paradoxus* (Naseeb *et al.* 2017b). Here, we reconstructed the phylogeny using a multigene concatenation approach, which combines many genes together giving a large alignment (Fitzpatrick *et al.* 2006; Brown *et al.* 2001; Baldauf *et al.* 2000). Combination of concatenated genes improves the phylogenetic accuracy and helps to resolve the nodes and basal branching (Rokas *et al.* 2003). To reconstruct the evolutionary events, we concatenated 101 universally distributed orthologs obtained from complete genome sequencing data (Table S7). Both novel strains were located in one single monophyletic group, with the *S. mikatae* (Figure 4). Since *S. jurei* also have a chromosomal translocation in common with *S. mikatae*, it further shows that the two species share similar evolutionary history and hence present in the same group on the phylogenetic tree.

### Introgression analysis

To determine whether the two *S. jurei* strains possessed any introgressed region from other yeast species, we compared *S. jurei* genome with those of *S. cerevisiae*, *S. mikatae*, *S. paradoxus* and *S. kudriavzevii*. We did not observe introgression of any full-length genes or large segments of the genome (>1000 bp) in *S. jurei*. However, in both novel strains, we identified seven small DNA fragments (300 bp-700 bp) belonging to five different genes, which may have derived from *S. paradoxus* or *S. mikatae* (Table S8). DNA fragments from all the genes (*CSS3*, *IMA5*, *MAL33*, *YAL003W*) with the exception of *YDR541C*, showed a high sequence similarity to *S. paradoxus* genome, indicating putative introgression from *S. paradoxus* to *S. jurei* (Table S8).

Introgression of genetic material can easily occur in *Saccharomyces* species by crossing the isolates to make intraspecific or interspecific hybrids (Fischer *et al.* 2000; Naumov *et al.* 2000). Among the *Saccharomyces sensu stricto* group, introgressions have been demonstrated in natural and clinical yeast isolates (Liti *et al.* 2006; Zhang *et al.* 2010; Wei *et al.* 2007; Muller and McCusker 2009) and in wine, beer and other fermentation environments (de Barros Lopes *et al.* 2002; Usher and Bond 2009; Dunn *et al.* 2012). It is generally believed that introgressed regions are retained, as they may be evolutionarily advantageous (Strope *et al.* 2015; Novo *et al.* 2009). Previous studies have demonstrated that introgression in *S. cerevisiae* is relatively common and a majority of the genes are derived from introgression with *S. paradoxus* (Strope *et al.* 2015; Warringer *et al.* 2011; Novo *et al.* 2009; Liti *et al.* 2006; Peter *et al.* 2018).

### Phenotypic profiling of *S. jurei*

We performed large-scale phenotypic profiling under various stress conditions and at different temperatures to capture the fitness landscape of *S. jurei* (strains NCYC 3947^T^ and NCYC 3962) relative to other *Saccharomyces sensu stricto* species. Type strains of all *Saccharomyces sensu stricto* species were used for fitness analysis. Colony size was taken as a proxy for fitness score (see methods). Generally the fitness of *S. jurei* NCYC 3962 in different environmental stressor conditions was higher compared to *S. jurei* NCYC 3947^T^ (Figure 3). Remarkably, only *S. jurei* NCYC 3962 was able to grow well on higher concentrations of acetic acid (Figure 3). Like most of the other *Saccharomyces* yeast species, both strains of *S. jurei* can also grow in media containing 10% ethanol. Although *S. eubayanus* showed the highest fitness in media containing 15% maltose, both strains of *S. jurei* were also able to tolerate high concentrations of maltose. Moreover, *S. jurei* NCYC 3962 was able to better tolerate higher concentrations of H_2_O_2_, CuSO_4_ and NaCl compared to most of the other *sensu stricto* species (Figure 3). *Saccharomyces* yeast species can acquire copper tolerance either due to an increase in *CUP1* copy number (Warringer *et al.* 2011) or due to the use of copper sulfate as a fungicide in vineyards (Fay *et al.* 2004; Perez-Ortin *et al.* 2002). The genomic analysis shows that both strains of *S. jurei* possess one copy of *CUP1*, indicating other factors maybe associated with copper tolerance.

Phenotypically, both strains of *S. jurei* clustered with *S. mikatae* and *S. paradoxus*, which is in accordance with our phylogenetic results, and, interestingly, the brewing yeast *S. eubayanus* was also present in the same cluster (Figure 3). This may indicate that in spite of the phylogenetic distance, *S. eubayanus* may have shared similar ecological conditions with the other above mentioned species.

We also evaluated the fitness of *S. jurei* strains in comparison to *Saccharomyces sensu stricto* species at different temperatures, taking into account growth parameters such as lag phase (***λ*)**, maximum growth rate (µ_max_), and maximum biomass (*A*_max_) (Tables S9-S11). The optimum growth of NCYC 3947^T^ and NCYC 3962 was at 25° and 30° respectively (Table S10). Both strains of *S. jurei* are able to grow at a high temperatures (*i.e.*, 37°) compared to *S. kudriavzevii*, *S. pastorianus*, *S. arboricola*, *S. uvarum*, and *S. eubayanus*, which are unable to grow at 37° (Table S10). The ability of *S. jurei* strains to grow well both at cold and warm suggest that this species evolved to be a generalist rather than a specialist in terms of thermoprofiles. The growth profiles captured at different temperatures for the other *Saccharomyces* species was in accordance to the previously published study (Salvadó *et al.* 2011).

### Conclusions

High quality *de novo* assembly of two novel strains of *S. jurei* (NCYC 3947^T^ and NCYC 3962) has been carried out using short and long reads sequencing strategies. We obtained a 12 Mb genome and were able to assemble full chromosomes of both strains. We found two reciprocal chromosomal translocations in *S. jurei* genome, between chromosomes I/XIII and VI/VII. The translocation between chromosomes I/XIII is unique to *S. jurei* genome, whereas the translocation between VI/VII is shared with *S. mikatae* IFO1815 and IFO1816. This suggests a common origin between *S. jurei* and *S. mikatae* and *S. jurei* evolved after acquiring the translocation between chromosomes I/XIII, while *S. mikatae* 1815 acquired a second translocation between chromosomes XVI/VII. Moreover, both strains of *S. jurei* showed low heterozygosis within themselves and were genetically diverged possessing 6227 SNPs between them. We found 4 novel ORFs that had no significant match to any of the available genomes. *S. jurei* genome had an increased number of Ty elements compared to *S. cerevisiae* and showed no signatures of introgression. The phylogenetic analysis showed that the novel species is closely related to *S. mikatae*, forming a single monophyletic group.

Phenotypically, the environmental stressor profiles of *S. jurei* are similar to those of with *S. mikatae*, *S. paradoxus*, *S. cerevisiae* (which further reiterate that *S. jurei* is closely related to these species) and *S. eubayanus*. We found that *S. jurei* NCYC 3962 compared to other *sensu stricto* species was able to grow well at high concentrations of acetic acid. In general, *S. jurei* NCYC 3962 showed relatively higher fitness compared to *S. jurei* NCYC 3947^T^ under most of the environmental stress conditions tested. Both strains of *S. jurei* showed similar growth rate at relatively low temperature, however, NCYC 3962 showed increased fitness compared to NCYC 3947^T^ at higher temperatures. The sequencing data and the large-scale phenotypic screening of this new species provide the basis for future investigations of biotechnological and industrial importance.
